# Retnla (Relmα/Fizz1) Suppresses Helminth-Induced Th2-Type Immunity

**DOI:** 10.1371/journal.ppat.1000393

**Published:** 2009-04-17

**Authors:** John T. Pesce, Thirumalai R. Ramalingam, Mark S. Wilson, Margaret M. Mentink-Kane, Robert W. Thompson, Allen W. Cheever, Joseph F. Urban, Thomas A. Wynn

**Affiliations:** 1 Laboratory of Parasitic Diseases, National Institute of Allergy and Infectious Diseases, National Institutes of Health, Bethesda, Maryland, United States of America; 2 Biomedical Research Institute, Rockville, Maryland, United States of America; 3 Diet, Genomics, & Immunology Laboratory, Beltsville Human Nutrition Research Center, Agricultural Research Service, United States Department of Agriculture, Beltsville, Maryland, United States of America; University of Wisconsin-Madison, United States of America

## Abstract

*Retnla* (Resistin-like molecule alpha/FIZZ1) is induced during Th2 cytokine immune responses. However, the role of Retnla in Th2-type immunity is unknown. Here, using Retnla^−/−^ mice and three distinct helminth models, we show that Retnla functions as a negative regulator of Th2 responses. Pulmonary granuloma formation induced by the eggs of the helminth parasite *Schistosoma mansoni* is dependent on IL-4 and IL-13 and associated with marked increases in Retnla expression. We found that both primary and secondary pulmonary granuloma formation were exacerbated in the absence of Retlna. The number of granuloma-associated eosinophils and serum IgE titers were also enhanced. Moreover, when chronically infected with *S. mansoni* cercariae, Retnla^−/−^ mice displayed significant increases in granulomatous inflammation in the liver and the development of fibrosis and progression to hepatosplenic disease was markedly augmented. Finally, Retnla^−/−^ mice infected with the gastrointestinal (GI) parasite *Nippostrongylus brasiliensis* had intensified lung pathology to migrating larvae, reduced fecundity, and accelerated expulsion of adult worms from the intestine, suggesting Th2 immunity was enhanced. When their immune responses were compared, helminth infected Retnla^−/−^ mice developed stronger Th2 responses, which could be reversed by exogenous rRelmα treatment. Studies with several cytokine knockout mice showed that expression of Retnla was dependent on IL-4 and IL-13 and inhibited by IFN-γ, while tissue localization and cell isolation experiments indicated that eosinophils and epithelial cells were the primary producers of Retnla in the liver and lung, respectively. Thus, the Th2-inducible gene Retnla suppresses resistance to GI nematode infection, pulmonary granulomatous inflammation, and fibrosis by negatively regulating Th2-dependent responses.

## Author Summary

Retnla is a member of a family of cysteine-rich secreted proteins, referred to as ‘resistin-like molecules’ or ‘found in inflammatory zone’ that increase in expression during allergic reactions and following infection with a variety of metazoan parasites. Retnla was originally hypothesized to function as an effector molecule during helminth-induced Th2-type immune responses. Studies conducted here with Retnla-deficient mice, however, suggest that Retnla primarily functions as a regulatory molecule during helminth infection. Using three helminth model systems affecting three different organ systems, we show that Retlna is induced by IL-4 and IL-13 as a mechanism to suppress Th2-type immunity. Retnla deficiency increased inflammation in the lung following i.v. challenge with *Schistosoma mansoni* eggs. Retnla deficiency also accelerated the development of liver fibrosis following *S. mansoni* infection. This finding was particularly surprising since Retnla was previously shown to activate collagen-producing fibroblasts that induce fibrosis. Thus, Retnla may represent a novel target for the treatment of fibrotic diseases. Finally, resistance to the intestinal nematode parasite *Nippostrongylus brasiliensis* was significantly increased in the absence of Retnla. When viewed together, the combined results from all three models establish a critical role for the Th2-inducible gene Retnla (Fizz1/Relm-alpha) in the suppression of helminth-induced Th2-type immunity.

## Introduction

Th1 and Th17 cytokines are believed to be proinflammatory, while Th2 cytokines were historically described as regulatory mediators [1],[2]. Now, however, it is well established that Th2 responses, characterized by the production of IL-4, IL-5, and IL-13, exhibit pleiotropic activities in host immunity [3]. In addition to their regulatory activities, they function as mediators of allergic inflammation, tissue remodeling, and fibrosis [4]. They also regulate immune homeostasis in the gut and promote resistance to GI nematodes [5],[6]. Indeed, polarized Th2 cytokine responses are a well-documented feature of most chronic helminth infections [7],[8]. Surprisingly, while the mechanisms that promote Th2 response development are being deciphered in many diseases, much less is known about the function of the downstream genes targeted by the Th2 effector response. Recently, we and others used oligonucleotide microarray analysis to characterize the global gene expression patterns associated with highly polarized Th1 and Th2 effector responses both in vitro and in vivo [9]–[12]. Among the list of unique Th2-inducible genes uncovered in these studies was Retnla (FIZZ1/Relma), which is induced to very high levels in a variety of tissues during Th2-polarized inflammatory responses [13],[14].

Retnla is a member of a family of cysteine-rich secreted proteins, referred to as ‘resistin-like molecules’ or ‘found in inflammatory zone’ originally identified in the lung [15]. The resistin-like family consists of four members, Retnla/Relma/FIZZ1, Relmb/FIZZ2, Resistin/FIZZ3, and Relmg/FIZZ4 [16]. Retnla is expressed in bronchial epithelial cells and in the wall of the large and small bowel and was originally hypothesized to be involved in the regulation of obesity and type-2 diabetes [17]. Retnla increases significantly during allergic responses in the lung and expression is IL-4/IL-13 and Stat6-dependent [14],[18],[19]. In addition to hypertrophic and hyperplastic bronchial epithelium, Retnla is also differentially expressed in alternatively- (AAM) and classically activated (CAM) macrophages, serving as a biomarker of AAM [20]–[22]. Despite its strong association with Th2 responses, however, the role of Retnla in Th2-driven immunity is unknown. Both the types of cells affected by and the responses to Retnla remain largely undefined. Recent studies suggested that Retnla is possibly involved in the induction of fibrosis in the lung by promoting the differentiation of myofibroblasts that mediate collagen deposition [18], [23]–[25]. Thus Retnla has been hypothesized to facilitate wound-repair and fibrosis at the site of Th2-mediated inflammatory responses [26]–[30].

In addition to being upregulated during Th2-associated pulmonary inflammation, Retnla is also found in abundance following infection with a variety of metazoan parasites [3], [13], [14], [31]–[33]. In schistosomiasis, development of liver fibrosis and portal hypertension is the primary cause of chronic morbidity and mortality, and Th2 cytokines have been shown to play a critical role [34]–[36]. Not surprisingly then, Retnla is markedly induced in the granulomatous tissues of *S. mansoni* infected and egg-challenged mice [13],[14]. Retnla is also upregulated following infection with *N. brasiliensis* [13],[27],[33]. Nevertheless, whether Retnla is directly involved in the regulation of Th2-dependent pathology or susceptibility to GI nematode infection remains unknown. A recent study showed that the related Retnla family member, Retnlb, could impair the chemosensory activity of the parasite *S. stercoralis* in vitro [37]. As such, these authors suggested that Retnlb serves as a direct Th2-dependent effector molecule (mediator of resistance) during GI nematode infection. Subsequent studies with Retnlb-deficient mice, however, demonstrated that the generation of protective Th2 cytokine responses and resistance to GI nematode infection was not dependent on Retnlb [38]. Thus, the functional contributions of the Th2 cytokine inducible Relm family during helminth infection and Th2 response development remain unclear.

To elucidate the physiological function of Retnla, mice with a targeted deletion of *Retlna* and insertion of the reporter LacZ were investigated [39],[40]. To determine whether Retnla was involved in the regulation of Th2 driven inflammation and fibrosis, Retnla knockout/reporter mice were compared with Retnla^+/+^ mice following both acute and chronic infection with *S. mansoni*. In addition, *S. mansoni* eggs were injected into the lungs to determine whether there were any tissue-specific differences in either the expression or activity of Retnla. Finally, studies were performed with the nematode *N. brasiliensis* to determine whether Retnla was required for the development of protective Th2 cytokine responses in the lung and intestine during nematode infection. Our results demonstrate that Retnla is not required for the development of helminth-induced Th2 responses in the lung, liver, or gut. Instead, Th2 dependent pulmonary inflammation, *S. mansoni*-induced liver fibrosis, and GI nematode expulsion were all significantly enhanced in the absence of Retnla. Thus, these studies demonstrate that Retnla, although induced by IL-4/IL-13, functions as a feedback mechanism to suppress Th2 responses. In the case of *S. mansoni*, Retnla deficiency triggered severe inflammation in the lung and liver, leading to the accelerated development of hepatosplenic disease following infection, while in the case of *N. brasiliensis*, Retnla^−/−^ mice expelled their parasites more rapidly from the gut. Thus, Retnla exhibits either protective or disease exacerbating activities by functioning as a negative regulator of Th2 responses.

## Results

### Expression of Relm family members and characterization of Retnla^−/−^ mice

**Figure 1:**
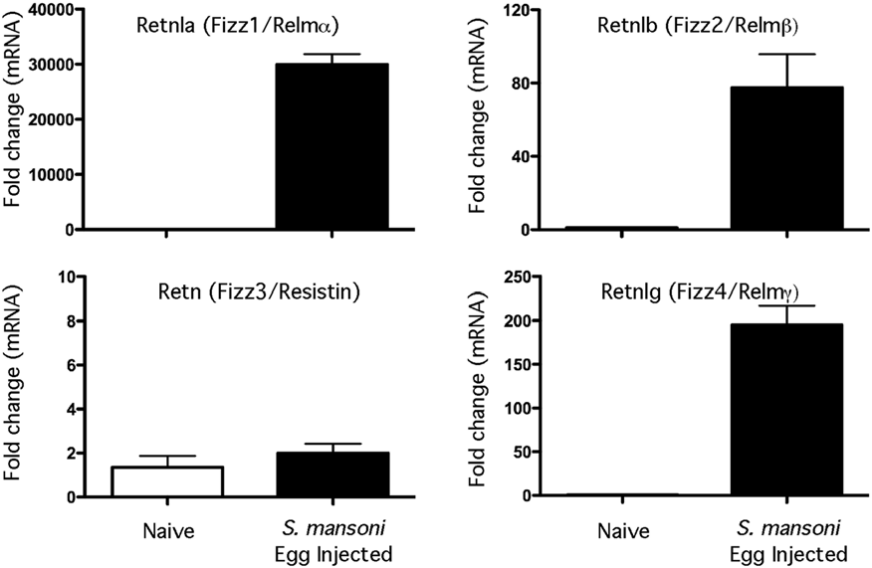
*Retnla, Retnlb*, and *Retnlg* expression is induced during Th2 inflammation. WT C57BL/6 (*n* = 5 per group) mice were sensitized i.p. with freshly isolated eggs of *S. mansoni* and challenged i.v. 14 days later. On day 7 after challenge mice were sacrificed and lung RNA specimens were prepared individually for real-time PCR analysis of Retnla, Retnlb, Retn, and Retnlg. Gene expression (mean±SEM) is expressed as the fold-increase over naïve WT controls after normalization to HPRT. Similar results were obtained in several repeat experiments.

To determine which members of the Relm gene family were most responsive to Th2 driven inflammation, 5000 viable *S. mansoni* eggs were injected intravenously into the lungs of C57BL/6 mice and expression of Retnla, Retnlb, Retn, and Retnlg mRNA was measured in the granulomatous tissues by quantitative real-time PCR. *S. mansoni* eggs have been shown in numerous studies to induce highly polarized Th2-dependent inflammatory responses by day 7 post-challenge [13],[34]. Development of the granulomatous response was associated with a significant increase in Retnla, Retnlb, and Retnlg mRNA (Fig. 1). However, Retnla was consistently upregulated to a much larger extent, often 100 times greater than Retnlb or Retnlg. In contrast, there was no increase of Retn (Resistin/FIZZ3) mRNA during the Th2-driven response. To elucidate the function of Retnla in the Th2 dependent inflammatory response, Retnla^−/−^ mice were generated at Regeneron Pharmaceuticals using VelociGene technology [39],[40]. The genotypes of Retnla^+/+^, Retnla^+/−^ and Retnla^−/−^ offspring were determined by PCR analysis (data not shown). Retnla^−/−^ mice were healthy and fertile and manifested no physical impairment. Naive Retnla^−/−^ mice also showed no fundamental anomalies in the lymphoid compartment (Fig. S1). CD4^+^, CD8^+^ and CD4^+^D8^+^ populations were present at the expected frequencies and numbers in the thymus. B cells, CD4^+^ and CD8^+^ T cells, and natural killer cells were also present in wild-type proportions and numbers in the spleen.

### Localization of Retnla expression during Th2-driven pulmonary inflammation

**Figure 2:**
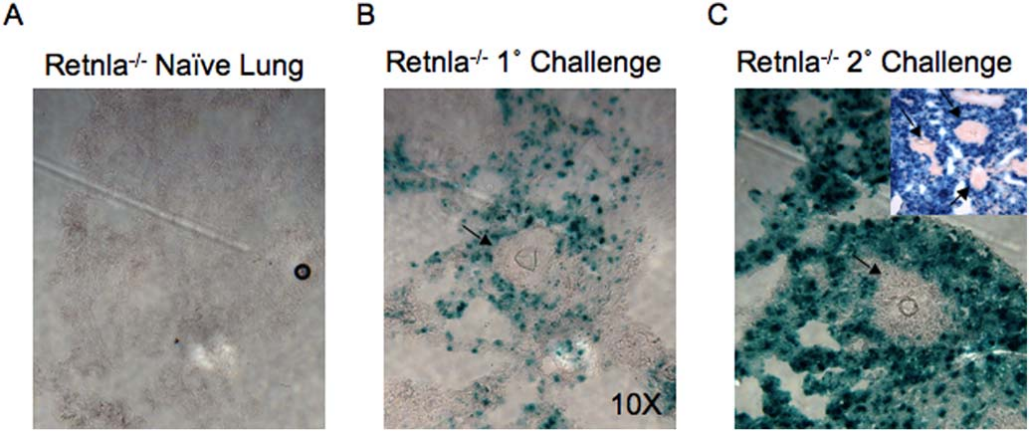
Retnla is localized to the lung parenchyma surrounding the granulomas. (A) Lung samples from naïve, (B) *S. mansoni* i.v. egg challenged (primary) and (C) i.p. egg sensitized and challenged (secondary) Retnla^−/−^ mice were snap frozen and embedded in OCT. In situ Retnla expression was evaluated by LacZ reporter activity (blue precipitate) in frozen tissue sections exposed to β-galactosidase substrate, X-gal. Original magnification ×20. Small inset in panel C magnification ×10.

Because the construction of the Retnla^−/−^ mouse included an in-frame insertion of a lacZ reporter gene, we examined β-galactosidase activity in the tissues as a surrogate of Retnla expression. As predicted from the mRNA results (Fig. 1), there was almost no positive β-gal staining in the lungs of naïve mice (Fig. 2A). Following the injection of eggs into the lungs of unsensitized (Fig. 2B) or egg-sensitized mice (Fig. 2C), however, significant β-gal activity was detected in the lung parenchyma, particularly in areas surrounding the granulomas. Retnla expression was strongest in the mice that were both sensitized and challenged with *S. mansoni* eggs (Fig. 2C), corresponding to the greatest Th2 response. Strikingly, β-gal staining was almost completely excluded from the granulomatous lesions where the greatest numbers of inflammatory cells were located (inset, Fig. 2C). This spatial pattern suggested that Retnla was predominantly derived from the lung parenchyma, most likely epithelial cells, rather than the eosinophil/macrophage-rich leukocytic infiltrate that dominates within the granulomas [41].

### Pulmonary granulomatous inflammation increases in Retnla^−/−^ mice

**Figure 3:**
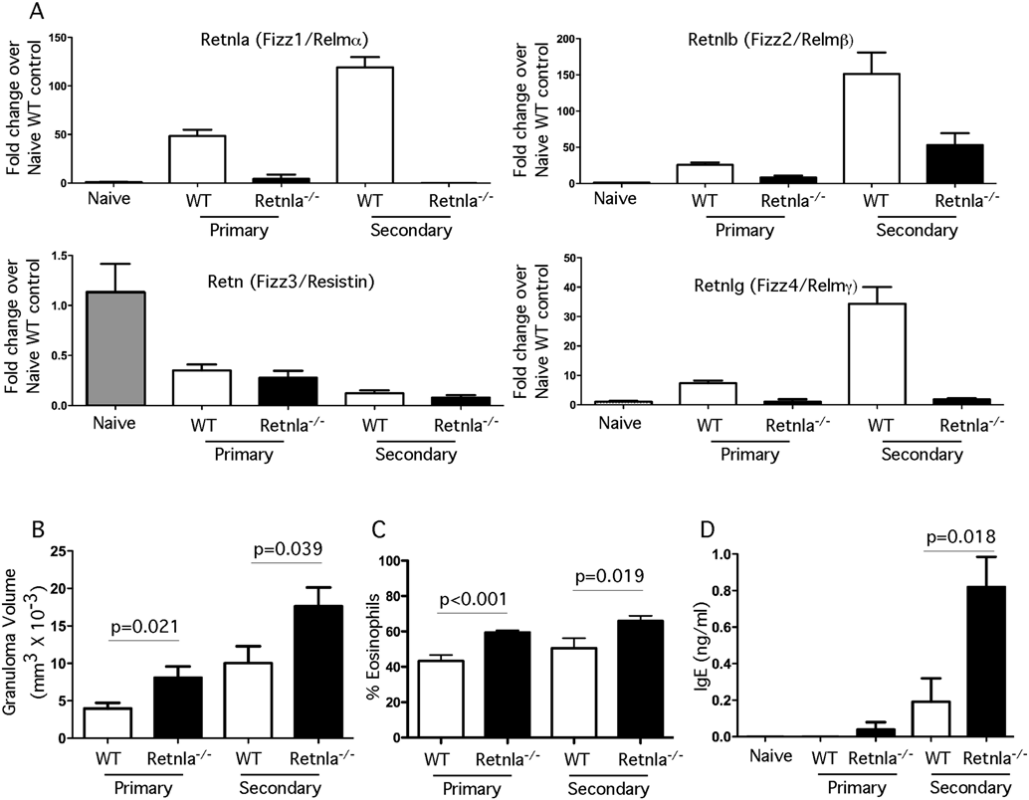
Pulmonary granuloma formation is increased in the absence of Retnla. Naïve, *S. mansoni* i.v. egg challenged (primary) and i.p. egg sensitized and challenged (secondary) WT and Retnla^−/−^ mice were sacrificed on day 7 post i.v. egg-challenge. Right lung RNA specimens were prepared individually for real-time PCR analysis of Retnla, Retnlb, Retn, and Retnlg. Primary WT (n = 9), Primary Retnla^−/−^ (n = 8), Secondary WT (n = 9), Secondary Retnla^−/−^ (n = 10). Gene expression (mean±SEM) is expressed as the fold-increase over naïve WT controls after normalization to HPRT (A). The left lobes from each mouse were examined histologically to evaluate the size of granulomas as a measure of inflammation (B) and the percentage of granuloma-associated eosinophils (C). Serum was also collected to quantify SEA-specific IgE (mean ng/ml±SEM) (D). Statistically significant differences and the corresponding p values are noted in the figures. Similar results were obtained in two repeat experiments.

Because schistosome eggs significantly increased Retnla expression (Fig. 1 and 2), we examined whether Retnla regulates the development of granuloma-associated inflammatory responses in the lung. For these studies, naïve and *S. mansoni*-egg sensitized mice were challenged intravenously with 5000 live *S. mansoni* eggs containing mature miracidia and primary and secondary granulomatous responses were measured in the lungs on day 7 post-challenge. As expected, *Retnla* mRNA was not detected in the lungs of naïve or egg-challenged Retnla^−/−^ mice, while WT mice showed a marked increase in *Retnla* expression following egg exposure in both the primary and secondary granuloma models (Fig. 3A). Unexpectedly, expression of *Retnlb* and *Retnlg* mRNA was markedly reduced in the Retnla^−/−^ mice, suggesting that Retnla is required for the production of the other inducible Relm family members. Strikingly, while expression of *Retnlb* and *Retnlg*, in addition to *Retnla*, were markedly reduced in the Retnla^−/−^ mice, primary and secondary granuloma formation both increased significantly in the absence of Retnla (Fig. 3B). The exacerbated granulomatous responses in the Retnla^−/−^ mice was associated with a marked increase in granuloma-associated eosinophils (Fig. 3C) and serum IgE titers (Fig. 3D). Retnla^−/−^ mice also displayed increased airway hyperreactivity when compared with WT mice (data not shown), suggesting that multiple Th2 -associated responses were enhanced in the lungs of Retnla^−/−^ mice.

### Retnla is expressed predominantly by granuloma eosinophils during infection

**Figure 4:**
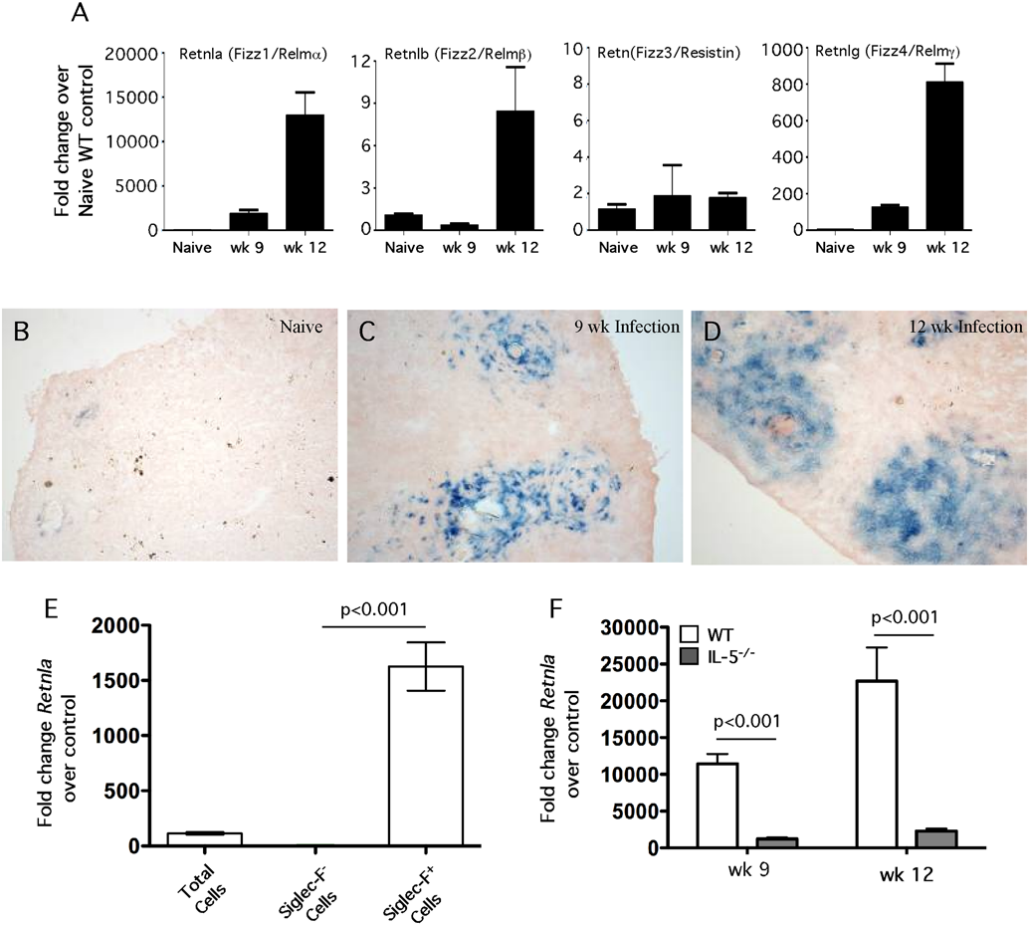
Retnla is localized to granuloma-associated eosinophils in the liver. Control WT C57BL/6 mice (*n* = 5) were infected with 30–35 *S. mansoni* cercariae percutaneously. Mice were sacrificed at weeks 9 and 12 post infection and liver RNA specimens were prepared individually for real-time PCR analysis of Retnla, Retnlb, Retn, and Retnlg (A). Gene expression (mean±SEM) is expressed as the fold-increase over naïve WT controls after normalization to HPRT. Livers from naïve Retnla^−/−^ mice (B) and Retnla^−/−^ mice infected with 30–35 cercariae for 9 (C) and 12 wk (D) were snap frozen and embedded in OCT. In situ Retnla expression was evaluated by LacZ reporter activity (blue precipitate) in frozen tissue sections exposed to β-galactosidase substrate, X-gal. Original magnification ×10 (B, C) and ×20 (D). (E) WT C57BL/6 mice were infected with 35 *S. mansoni* cercariae and on week 9, eosinophils were purified from perfused livers by positive selection using the Siglec-F antibody. The granuloma-associated leukocytes were separated into nonfractionated, Siglec-F^−^ (eosinophil-negative), and Siglec-F^+^ (eosinophil-positive) fraction, mRNA was isolated, and then analyzed by real-time PCR analysis for *Retnla*. The data shown are means±SEM (*n* = 5/group). (F) WT C57BL/6 mice and IL-5^−/−^ mice were infected with 30–35 *S. mansoni* cercariae percutaneously. Mice were sacrificed at weeks 9 (WT *n* = 9, IL-5^−/−^ *n* = 10) and 12 (WT *n* = 6, IL-5^−/−^ *n* = 8) post infection and liver RNA specimens were prepared individually for real-time PCR analysis of Retnla. Gene expression (mean±SEM) is expressed as the fold-increase over naïve WT controls after normalization to HPRT. Similar results were obtained in two repeat experiments.

To determine whether Retnla is expressed during chronic Th2-dominated responses, we infected mice percutaneously with *S. mansoni* cercariae and analyzed their pathological reactions at both acute (9 wk, 3–4 wk after egg laying commences) and chronic time points (≥12 wk) following infection. As observed in the pulmonary granuloma studies, there was a marked upregulation of *Retnla*, *Retnlb*, and *Retnlg* mRNA expression in the livers of infected C57BL/6 mice, with peak expression for all three genes peaking at 12 wk after infection (Fig. 4A). In contrast to the other Relm family members, *Retn* was not induced in the liver during *S. mansoni* infection. In addition, as predicted from the mRNA results, there was little to no positive Retnla protein expressed (β-galactosidase staining) in the livers of naïve mice (Fig. 4B). Following infection, however, the Retnla reporter mice showed significant β-gal activity in the liver at both 9 (Fig. 4C) and 12 wk (Fig. 4D). In contrast to the lung granuloma studies where β-gal activity was localized to the lung parenchyma in areas outside the granulomas (Fig. 2C), staining in infected livers was concentrated in the egg-induced lesions where inflammatory cells were located (Fig. 4C and 4D).

Macrophages and eosinophils are believed to be the primary hematopoietic sources of Retnla [42]. To identify the dominant cellular source of Retnla in the hepatic granulomas, the leukocyte population was isolated from the granulomatous lesions of 8–9 wk infected C57BL/6 mice. The leukocytes were separated into eosinophil-positive and -negative fractions by column purification. Flow cytometry demonstrated that the purity of the positive fraction was approximately 95% eosinophils [36]. Morphological analysis with the fast green stain confirmed the identity of the purified population as eosinophils (data not shown). mRNA was isolated from eosinophil-negative and -positive fractions and then subjected to real-time PCR analysis. *Retnla* mRNA increased nearly 100-fold in the unfractionated cells (Fig. 4E). However, *Retnla* mRNA was enriched an additional 15-fold in the eosinophil positive fraction and correspondingly reduced to near background levels in the eosinophil negative population, which contained T cells, B cells, macrophages, and neutrophils. Finally, to confirm the association of Retnla with eosinophils, we infected WT C57BL/6 and IL-5^−/−^ mice with *S. mansoni* and then examined the expression of Retnla mRNA in the liver at 9 and 12 wk post-infection (Fig. 4F). As expected, the granulomas in the IL-5^−/−^ mice were nearly devoid of eosinophils at both time points (<1.0% of all cells), while the granulomas in the WT mice were composed of approximately 50% eosinophils [36]. Interestingly, even though the number of granuloma-associated macrophages increased from approximately 20% in the WT granulomas to more than 40% in IL-5^−/−^ mice [36], expression of *Retnla* was reduced to near background levels in the eosinophil-deficient mice (Fig. 4F). Together, these findings suggested that eosinophils were the dominant source of Retnla in the granulomatous livers.

### Retnla suppresses hepatic granuloma formation and fibrosis

**Figure 5:**
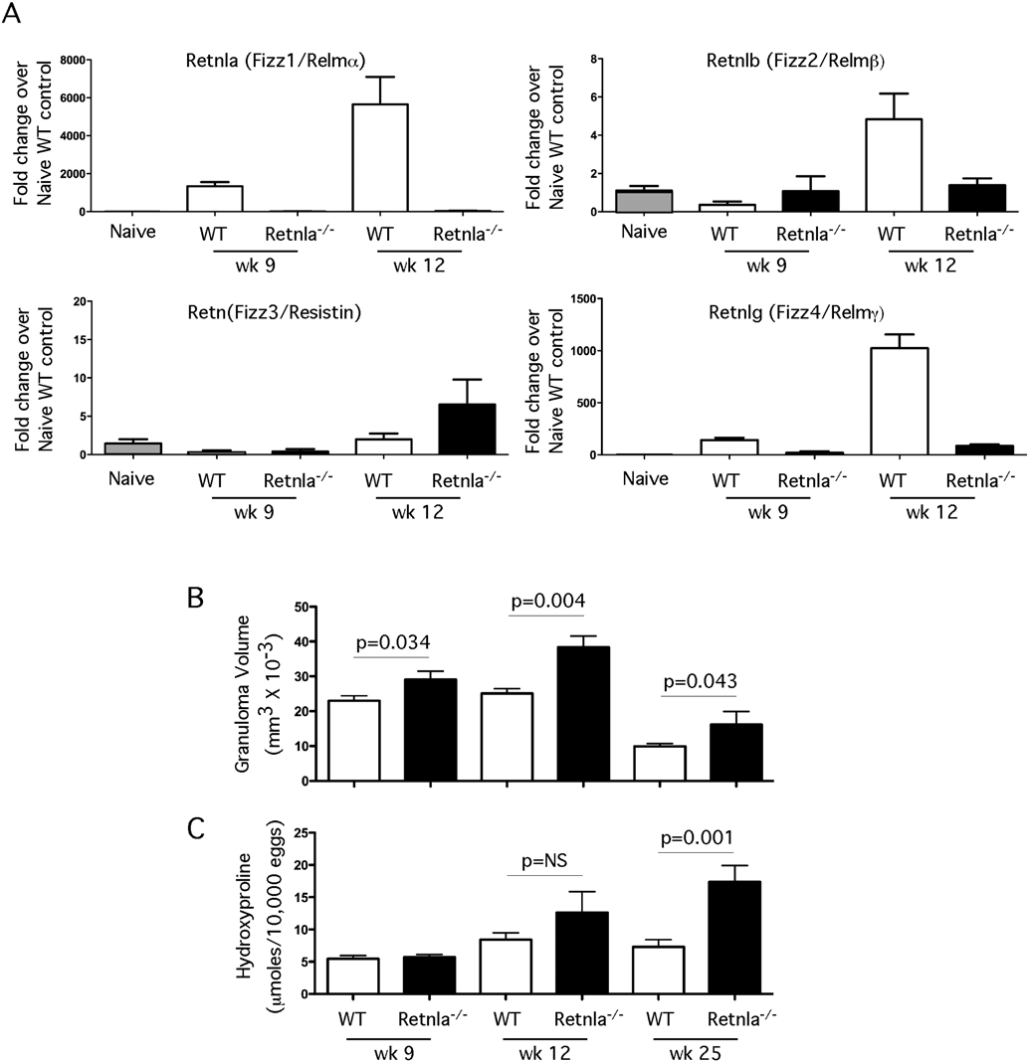
Inflammation and fibrosis are exacerbated in *S. mansoni* infected Retnla^−/−^ mice. (A) Control WT C57BL/6 mice (N = 19 wk 9, N = 7 wk 12) and Retnla^−/−^ (N = 17 wk 9, N = 8 wk 12) mice were infected with 30–35 *S. mansoni* cercariae percutaneously. Mice were sacrificed at weeks 9 and 12 post infection and liver RNA specimens were prepared individually for real-time RT-PCR analysis of Retnla, Retnlb, Retn, and Retnlg. Gene expression (mean±SEM) is expressed as the fold-increase over naïve WT controls after normalization to HPRT. In separate studies, the livers of *S. mansoni* infected mice were analyzed on wk 9 (WT N = 19, KO N = 17), 12 (WT N = 7, KO N = 8), and 25 (WT N = 10, KO N = 8) to evaluate the size of granulomas (Mean granuloma volume±SEM) as a measure of inflammation (B) and hydroxyproline content as a measure of fibrosis (C). Fibrosis was measured by quantifying hydroxyproline levels in the tissues and normalizing to the numbers of eggs deposited in the liver. Statistically significant differences and the corresponding p values are noted in the figures. Similar results were obtained in three separate experiments.

Next, we examined whether Retnla regulates Th2-dependent granuloma formation and fibrosis in the liver during chronic *S. mansoni* infection. For these studies, WT and Retnla^−/−^ mice were again infected percutaneously with *S. mansoni* cercariae and granuloma formation was compared at 9 and 12 wk. *Retnla* mRNA expression was undetectable in the livers of Retnla^−/−^ mice both before and after infection, while WT mice displayed a significant increase in *Retnla* mRNA at both the 9 and 12 wk time points (Fig. 5A). As observed in the lung, expression of *Retnlb* and *Retnlg* mRNAs were also reduced in the Retnla^−/−^ mice, particularly at the 12 wk time point, confirming that Retnla regulates the expression of the other inducible Relm family members. When the granuloma-associated inflammatory response was compared, the Retnla^−/−^ mice also displayed a marked and highly significant increase in granuloma size (Fig. 5B). Although the greatest increase in granuloma size was observed at the 12 wk chronic time point, the inflammatory response in the Retnla^−/−^ mice remained significantly elevated as late as 25 wk post-infection. The exacerbated granulomatous response in the Retnla^−/−^ mice was associated with a marked increase in hepatic fibrosis, particularly in chronically infected mice where collagen deposition, assessed by hydroxyproline content, increased nearly 60% by wk 25 when compared with infected WT mice (Fig. 5C). Importantly, the increase in granuloma size and fibrosis in the Retnla^−/−^ mice was not associated with any significant change in the infectious burden, as assessed by the worm and tissue egg counts determined at 9, 12, and 25 wk post-infection (Table 1). Thus, the exacerbated disease in the infected Retnla^−/−^ mice was not the result of enhanced susceptibility to *S. mansoni* infection.

**Table 1:** Parasite burdens in wild type versus Retnla^−/−^ mice at 9, 12, and 25 wk post *S. mansoni* infection.

Strain	Week	Worm Pairs	Males	Females	Eggs/Worm Pair (1000 s)
Wild type	9	4.66±1.35	3.00±1.22	0.20±0.19	8.29±0.72
Retnla^−/−^	9	4.19±0.57	2.55±0.77	0.11±0.10	7.06±1.01
Wild type	12	3.57±0.60	0.00±0.00	1.42±0.44	13.82±1.72
Retnla^−/−^	12	4.37±0.66	0.25±0.23	0.37±0.17	11.28±1.78
Wild type	25	3.00±0.42	0.82±0.40	0.64±0.43	22.54±5.19
Retnla^−/−^	25	2.90±0.36	1.90±0.48	0.10±0.09	25.70±4.10

Means±SEM with approximately 10 mice per group at each time point. No significant differences in worm pairs or eggs/worm pair were noted between WT and Retlna^−/−^ mice at any time point.

### Retnla^−/−^ mice display increased resistance to *Nippostrongylus brasiliensis*

**Figure 6:**
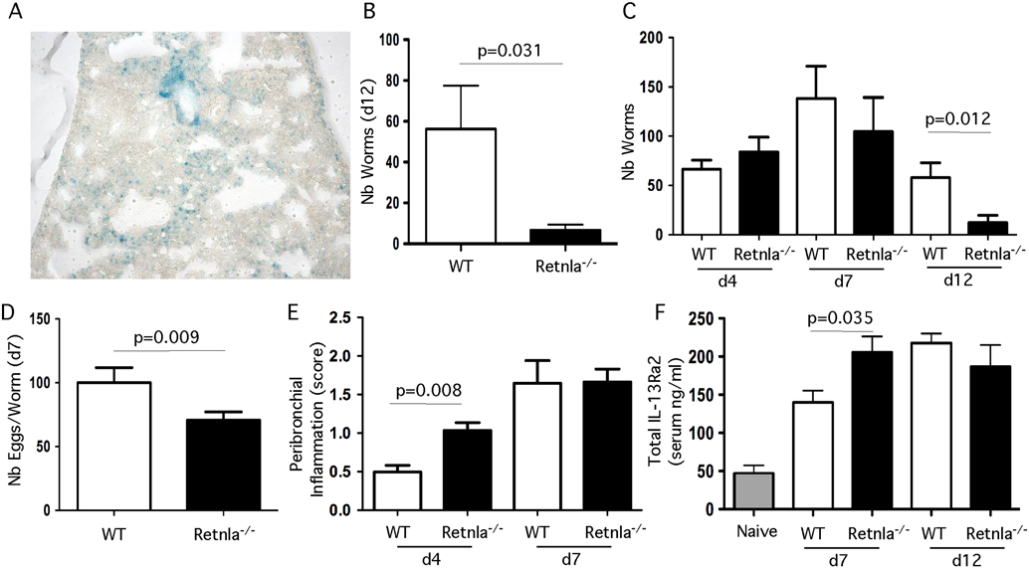
Retnla^−/−^ mice are more resistant to *Nippostrongylus brasiliensis* infection. (A) Lung samples from Retnla^−/−^ mice infected with 500 *N. brasiliensis* larvae (L3) were snap frozen and embedded in OCT. In situ Retnla expression was evaluated by LacZ reporter activity (blue precipitate) in frozen tissue sections exposed to β-galactosidase substrate, X-gal. Original magnification ×20. (B) Analysis of control WT (N =  5) and Retnla^−/−^ (N = 6) mice inoculated subcutaneously with 500 *N. brasiliensis* L3 and killed on day 12 after infection to assess adult worm recovery. (C) Control WT (N = 5, d4 and d7 and N = 10, d12) and Retnla^−/−^ (N = 7, d4, N = 5, d7, and N = 10, d12) mice infected with 500 *N. brasiliensis* L3 larvae and killed on day 4, 7, and 12 after inoculation to assess adult worm recovery. (D) *N. brasiliensis* fecal eggs/worm day 7 post-infection (N = 5 each). (E) Peribrochiolar inflammation was scored on day 4 (WT N = 5, KO N = 7) and 7 (N = 5) post-infection (score 1–4). (F) Serum was isolated on day 7 and 12 post-inoculation and the amount of sIL-13Rα2 was quantified by ELISA in WT (N = 5) and Retnla^−/−^ mice (N = 5) and reported as the average±SEM. Statistically significant differences and the corresponding p values are noted in all relevant figures. Similar results were obtained in two separate experiments.

To determine whether Retnla was required for the development of immunity in the gut, we infected Retnla^−/−^ mice with the GI nematode parasite *N. brasiliensis*. In this model, infective third-stage larvae are injected subcutaneously, migrate through the lungs, and mature in the jejunum 5–6 d after inoculation [7]. The majority of the adult parasites are expelled from the intestine 12 to14 days post-inoculation in C57BL/6 mice. As observed with *S. mansoni* eggs, *N. brasiliensis* induced significant Retnla expression in the lungs (Fig. 6A). Interestingly, the Retnla^−/−^ mice appeared to be much more resistant to infection, with the majority of the Retnla^−/−^ mice expelling nearly all adult worms from the intestine by day 12 (Fig. 6B). To determine whether the Retnla^−/−^ mice were either more resistant to infection or simply clearing their parasites more rapidly from the intestine, parasite burden in the intestine was enumerated on days 4, 7 and 12 post-inoculation. These studies showed that the WT and Retnla mice were equally susceptible to *N. brasiliensis* infection. However, based on both worm (Fig. 6C) and fecal egg counts (Fig. 6D), the Retnla^−/−^ mice developed resistance more rapidly and the worms were less fecund than in WT mice. The increased resistance of Retnla^−/−^ mice was also associated with a more robust inflammatory response in the lungs on day 4 (Fig. 6E) and more rapid rise in the serum titers of sIL-13Rα2 (Fig. 6F). Because production of the soluble IL-13Rα2 is IL-4Rα-, IL-13Rα1-, and Stat6-dependent [4], these data combined with the schistosomiasis studies suggested that the Retnla^−/−^ mice were developing stronger IL-4/IL-13 responses following infection.

### Retnla is induced by IL-4/IL-13 and negatively regulates Th2 Immunity

**Figure 7:**
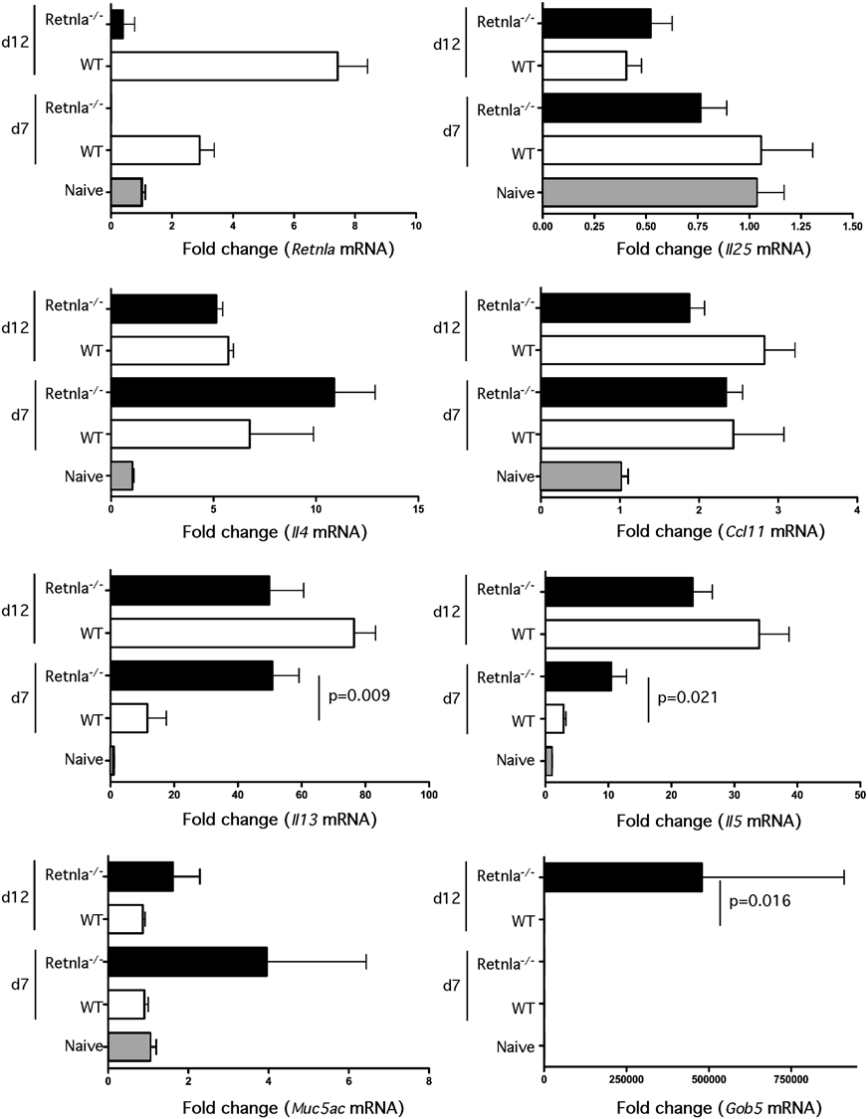
Th2 cytokine responses increase in *N. brasiliensis*-infected Retnla^−/−^ mice. WT (N = 5/time point) and Retnla^−/−^ (N = 5/time point) mice were inoculated subcutaneously with 500 *N. brasiliensis* L3 and the lungs were removed on day 7 and 12 after infection. The lungs were analyzed individually for *Retnla*, *Il25*, *Il4*, *Ccl11* (Eotaxin), *Il13*, *Il5*, *Muc5ac*, and *Gob5* mRNA by real-time quantitative PCR. Fold changes (Mean±SEM) are based on comparisons of infected mice with naive mice. Statistically significant differences and the corresponding p values are noted in the figures. Similar results were obtained in two separate experiments.

**Figure 8:**
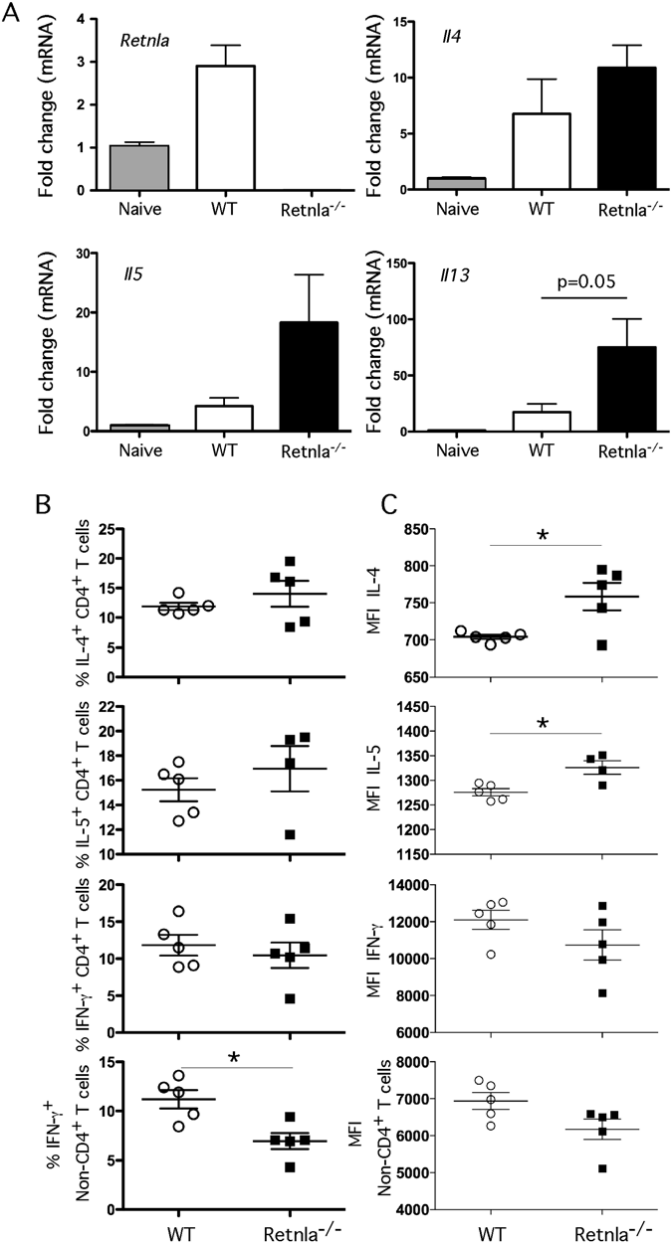
Retnla suppresses *S. mansoni* egg-induced Th2 responses. (A) Naïve and i.p. egg sensitized and challenged (secondary) WT (N = 5/time point) and Retnla^−/−^ (N = 5/time point) mice were sacrificed on day 7 post i.v. egg-challenge. Right lobe RNA specimens were prepared individually for real-time PCR analysis of *Retnla*, *Il4*, *Il5*, *and Il13*. Gene expression (Mean±SEM) is expressed as the fold-increase over naïve WT controls after normalization to HPRT. Statistically significant differences and the corresponding p values are noted in the panels. (B) Lung leukocytes were isolated, separated, counted and cultured with PMA, Ionomycin and Brefeldin A for 3 hrs and analyzed for ex-vivo cytokine production. Total number of IL-4, IL-5, and IFN-γ producing CD4^+^ T cells in the lung leukocyte preparation are shown for individual mice. The number of IFN-γ producing non-CD4^+^ T cells was also included (bottom panel). (C) Magnitude of cytokine production by CD4^+^ T cells displayed as the geometric mean of fluorescence intensity. Statistically significant differences are shown. All experiments were repeated twice with similar results.

Following infection with *N. brasiliensis*, the parasites migrate from the site of inoculation and enter the lungs via the circulatory system. Once inside the lungs, the parasites trigger a marked and highly polarized Th2 response, which was confirmed by analyzing the expression of several Th2-associated genes in the lungs (Fig. 7). WT mice displayed significant increases in *Retnla*, *Il4*, *Il5*, *Il13*, and *Ccl11* (Eotaxin) mRNA expression following infection with *N. brasiliensis*, while *Il25* and the mucin genes *Muc5ac* and *Gob5* were not altered. Strikingly, when the cytokine responses were compared, the Retnla^−/−^ mice showed highly significant increases in *Il5* and *Il13* on day 7 and non-significant increases in *Il4* mRNA than WT mice. In contrast to WT controls, we also observed marked increases in *Muc5ac* and *Gob5* in the Retnla^−/−^ mice (Fig. 7), suggesting that Th2 cytokine and Th2 effector molecules were enhanced in the absence of Retnla. The earlier and more marked increase in IL-13 expression in the Retnla^−/−^ mice likely explains their enhanced immunity to *N. brasiliensis*, as IL-13 is the crucial mediator controlling resistance to infection [7]. Similar increases in Il4, Il5, and Il13 mRNA expression were observed in the lungs of Retnla^−/−^ mice in response *S. mansoni* eggs (Fig. 8A). Lymphocytes were also isolated from the granulomatous lungs on day 8 after i.v. egg injection and intracellular cytokine staining was performed for IL-4, IL-5, and IFN-γ. Surprisingly, the number of IL-4-, IL-5-, and IFN-γ-producing CD4^+^ T cells in the lungs of Retnla^−/−^ mice was similar to WT mice during the secondary granulomatous response (Fig. 8B). However, the number of non-CD4^+^ IFN-γ producing cells decreased significantly in the Retnla^−/−^ mice (Fig. 8B, bottom panel), while the mean fluorescent intensity (MFI) for IL-4 and IL-5 increased (Fig. 8C), suggesting that the KO CD4^+^ T cells were producing more Th2 cytokines on a per cell basis.

**Figure 9:**
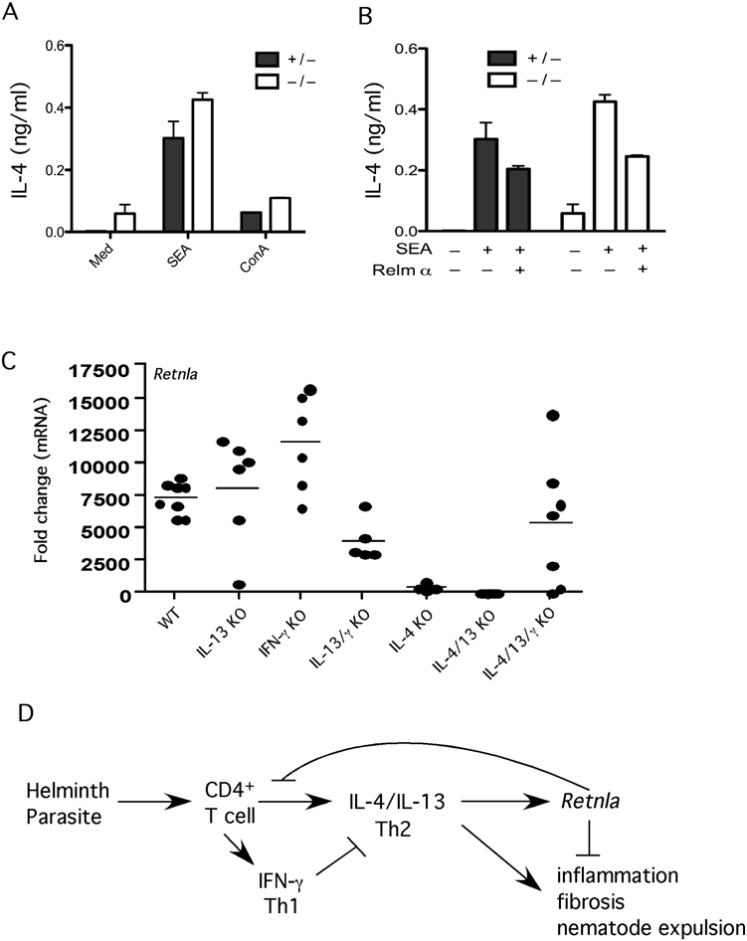
Retnla is induced by IL-4/IL-13 and inhibited by IFN-γ. (A) Splenocytes were harvested from mice sufficient (HET, +/−) or deficient (KO, −/−) in Retnla 12 days after i.p. sensitization with 5000 viable *S. mansoni* eggs. Cells were stimulated with media, SEA (25 µg/ml) or ConA (1 µg/ml) in duplicate for 72 h at 37°C at which time supernatants were harvested and tested for IL-4 by ELISA. (B) Separate groups of SEA stimulated cells were also treated with PBS or rRelma (0.5 µg/ml) and examined for IL-4 (bars show Means±SD). (C) WT C57BL/6, IL-13KO, IFN-γ KO, IL-13/IFN-γKO, IL-4KO, IL-4/IL-13KO, and IL-4/IL-13/IFN-γ KO mice were infected with 30–35 *S. mansoni* cercariae and liver mRNA was isolated on wk 9 post-infection and prepared individually for real-time RT-PCR analysis of *Retnla*. Gene expression (mean±SEM) is expressed as the fold-increase over naïve WT controls after normalization to HPRT. The results from individual mice are shown. (D) Model depicting the role of Retnla in the regulation of Th2-driven inflammation, fibrosis and immunity to GI nematode infection. Retnla is induced by IL-4 and IL-13 and inhibits the Th2 effector response.

Because Retnla deficiency also resulted in reduced expression of Retnlβ and Retnlγ (Fig. 3 and 5), it was unclear whether the observed increases in Th2 cytokine production were due specifically to the absence of Retnla. Therefore, to determine whether exogenous rRetnla (Relma) treatment could reverse the phenotype of the Retnla^−/−^ mice, we isolated splenocytes from Retnla^−/−^ mice and their heterozygous littermates following injection of *S. mansoni* eggs and measured the production of IL-4. As expected, the Retnla^−/−^ mice developed stronger IL-4 responses than control mice at baseline and following stimulation with SEA or ConA (Fig. 9A). However, when the splenocytes from the Retnla^−/−^ mice were treated with rRelmα, their SEA-stimulated IL-4 responses were returned to WT levels (Fig. 9B). These studies suggest that Retnla has a direct role in suppressing Th2 cytokine responses.

Finally, since Retnla was strikingly upregulated during immune responses to parasites, we dissected what type of immune response was required for the induction of Retnla. Mice genetically deficient in IL-4, IL-13, or IFN-γ alone or in combination were infected with *S. manson*i cercariae and expression of Retnla was measured in the liver by quantitative RT-PCR 9 wk post-infection (Fig. 9C). We found that Retnla mRNA was enhanced in the absence of IFN-γ and blocked or inhibited in the absence of IL-4 and IL-13, respectively. These combined results suggest that Retnla is preferentially induced by Th2 cytokines as a feedback mechanism to suppress the Th2 response. Consequently, in the case of *S. mansoni* infection, Retnla-deficiency leads to the development of more severe Th2-driven pathology, while in the case of *N. brasiliensis* infection the increased IL-13 response leads to increased pathology in the lungs, greater stress on the worm indicated by reduced fecundity, and the more rapid expulsion of the parasites from the gut (Fig. 9D).

## Discussion

‘Resistin-like molecules’ also known as ‘found in inflammatory zone’ proteins were originally identified in the lung and gut and expression of at least three of the family members [15], including Retnla, is strongly associated with the development of polarized Th2 responses [14],[21],[22]. Although Retnla was originally described as an inducible product of bronchial epithelial cells, intestinal crypt epithelium, and white adipose tissue [15],[43], Retnla is also expressed by eosinophils and is associated with the development of alternatively activated macrophages (AAMs) [21],[33],[39],[44]. Nevertheless, while it routinely used as a ‘marker’ of AAMs and Th2 responses more generally [19],[45], the functional role of Retnla was unknown. Using Retnla^−/−^ mice and several models of Th2-driven inflammation, we now demonstrate that the primary function of Retnla is to serve as a negative regulator of helminth-induced Th2-type immunity.

Studies with the helminth egg-induced granuloma model showed that Retnla expression was strongly dependent on the Th2 cytokines IL-4 and IL-13 and negatively regulated by IFN-γ. Thus, as observed in a few related Th2 models [27],[28],[33], Retnla expression was strongly dependent on the development of a polarized Th2-type response. Surprisingly, while studies with several helminths suggested that Retnla-expression was predominantly associated with the development of AAMs [13],[27],[32], our initial studies conducted with Retnla^−/−^ mice indicated that the majority of *S. mansoni* egg-induced Retnla expression was restricted to the lung parenchyma outside the granulomas. Although it seems likely that Retnla was also being produced by at least a subset of Th2-activated macrophages in this model, we found comparatively little Retnla expression in the pulmonary granulomas where the majority of AAMs are located. This expression pattern was also observed following *N. brasiliensis* infection. Thus, in these acute Th2 disease models, Retnla appeared to be produced predominantly by lung epithelial cells and much less so by recruited inflammatory cells like macrophages. These findings confirm earlier studies, which suggested epithelial tissues might be the dominant source of Retnla in the lung [15].

In the schistosome pulmonary granuloma model, studies conducted with IL-4^−/−^, IL-13^−/−^, and IL-4^−/−^/IL-13^−/−^ double knockout mice demonstrated that egg-induced granuloma formation is dependent on both IL-4 and IL-13. Indeed, the depletion of either cytokine alone reduced granulomatous inflammation by half, while the simultaneous depletion of both IL-4 and IL-13 nearly ablated granuloma development [46],[47]. Expression of Retnla was also reduced to near baseline levels in *S. mansoni* infected IL-4^−/−^/IL-13^−/−^ mice, suggesting that Retnla is an important product of the IL-4/IL-13 effector response. To determine why Retnla might be important, we challenged naïve and egg sensitized Retnla^−/−^ mice intravenously with live schistosome eggs and found that primary and secondary granuloma formation was markedly increased in the lungs. Because IL-4, IL-5, and IL-13 production also increased in the egg-challenged Retnla^−/−^ mice, the enhanced Th2 response provided the underlying mechanism for the exacerbated granulomatous response. We also observed an increase in the number of granuloma-associated eosinophils and the titers of SEA-specific IgE, providing further proof that the Retnla^−/−^ mice were generating stronger Th2 responses. Thus, instead of promoting Th2 effector function, the combined results from our pulmonary studies provided the first evidence that Retnla was inhibiting Th2 responses.

In marked contrast to the egg-induced lesions in the lung, Retnla was expressed within the liver granulomas of *S. mansoni* infected mice. Indeed, staining for Retnla was primarily localized to the granuloma-associated leukocytes, suggesting AAMs and/or other hematopoietic cells like eosinophils were likely the dominant source of Retnla in the infected liver. Cell isolation studies suggested that eosinophils were the dominant source. Given that Retnla was localized to different cellular compartments in the lung and liver, we examined whether Retnla was also modulating the development of egg-induced pathology during *S. mansoni* infection. While the dominant cellular source of Retnla differed between organs the effects of Retnla deficiency on the granulomatous response was similar in the lung and liver. Not only were the hepatic granulomas in the Retnla^−/−^ mice larger at the acute time point (wk 9) they remained significantly larger than WT granulomas even after 25 weeks of infection. The chronically infected Retnla mice also displayed significant increases in hepatic fibrosis, reaching levels that were nearly 60% greater than WT mice. Given that granuloma formation and hepatic fibrosis are highly dependent on IL-13 [48],[49], these data demonstrated that Retnla plays a major suppressive role in both acute and chronic Th2-driven pathological responses.

Retnla modulation of fibrosis was particularly unexpected, as numerous recent reports indicated that Retnla might be important for the development of fibrosis [23],[25],[29],[50]. Liu and colleagues showed that Retnla is one of the most highly upregulated genes in the bleomycin model of pulmonary fibrosis [24]. They found that Retnla expressing type II alveolar epithelial cells could stimulate alpha-smooth muscle actin and type I collagen expression in fibroblasts in vitro. Similar to our findings, they determined that Retnla was primarily expressed in airway epithelial cells in response to IL-4 and IL-13 [18]. They also showed that bleomycin-induced fibrosis was dependent on IL-4 and IL-13. Based on these observations, they suggested that IL-4/IL-13-induced Retnla was playing a direct role in myofibroblast differentiation and in the pathogenesis of pulmonary fibrosis. Nevertheless, the results from our studies demonstrated that Retnla plays a suppressive role in IL-4/IL-13-dependent fibrosis. The Retnla^−/−^ mice developed more severe fibrosis presumably because production of the profibrotic cytokines IL-4 and IL-13 increased significantly [48],[49],[51]. Importantly, the number of worms and eggs in the tissues did not differ significantly between WT and Retnla^−/−^ mice, indicating that the increase in fibrosis following *S. mansoni* infection was not due to changes in the parasite burden but rather resulted from the stronger granuloma-associated inflammatory response. Thus, instead of inducing fibrosis, these data demonstrate that Retnla is involved in the prevention or resolution of IL-4/IL-13-dependent fibrosis.

Given that Retnla exhibited suppressive activity in both lung and liver, we subsequently examined whether Retnla was modulating Th2 responses in the gut following nematode infection. Numerous studies with a variety of helminth parasites have shown that immunity to GI nematode infection is highly dependent on the induction of Th2-polarized responses [7] and recent studies have associated parasite resistance with significant Retnla and Retnlb expression in the tissues [21],[27],[33],[52],[53]. Artis and colleagues recently suggested that the related Relm family member, Retnlb, might serve as a general Th2-dependent effector molecule during GI nematode infection [37]. However, subsequent studies with Retnlb-deficient mice argued against a direct role for Retnlb in resistance to GI nematodes that invade the large intestine since WT and Retnlb^−/−^ mice were equally resistant to *Trichuris muris* infection [38]. Since immunity to the related gastrointestinal nematode parasite *N. brasiliensis* is also highly dependent on IL-4R/Stat6/IL-13-dependent signaling [7],[54], we examined whether resistance to nematode infection in the small intestine was affected by Retnla deficiency. In agreement with the results with Retnlb^−/−^ mice infected with *T. muris*, immunity to a nematode infection in the small intestine was not dependent on Retnla. In fact, the Retlnla^−/−^ mice expressed a more intense response to migrating larvae in the lungs, reduced the vigor of adults in the small intestine indicated by reduced fecundity, and expelled adult worms significantly faster, suggesting that Retnla was inhibiting the intensity of Th2-dependent immunity in the lungs and intestine. This conclusion was consistent with the increased IL-13 response observed in the tissues of Retlnla^−/−^ mice. The identification of an anti-inflammatory role for Retnla in the *N. brasiliensis* model was unexpected since a recent study using the dextran sodium sulfate (DSS) model of colonic inflammation suggested that Retnla plays a proinflammatory role in the gut [39]. Nevertheless, inflammation in the DSS model is driven by IL-12 and IFN-γ [55], while *N. brasiliensis*-infection is associated with the development of polarized Th2 responses. Consequently, by promoting Th1- and suppressing Th2-driven inflammation, Retnla appears to exhibit divergent activity during Th1- and Th2-polarized immune responses.

Thus, while Retnla was originally hypothesized to function as an important effector molecule during Th2 polarized immune responses [4],[24],[44], our studies with Retnla^−/−^ mice strongly suggest that Retnla primarily functions as a regulatory molecule. Using three separate model systems affecting three different tissues, we show that Retlna is induced by IL-4/IL-13 as feedback mechanism to suppress Th2 immunity. In the case of granuloma formation in the lung, the deficiency in Retnla led to the development of much larger granulomas and enhanced eosinophil and IgE responses. Thus, Retnla regulates several features that are commonly associated with allergic inflammation. Preliminary results with a murine asthma model suggest that Retnla also suppresses the development of AHR, which is consistent with the suppression of IL-13 responses by Retnla [56]. Retnla deficiency also significantly augmented the development of IL-13-dependent fibrosis following *S. mansoni* infection. Thus, Retnla may represent a novel target for the treatment of fibrotic diseases, which currently lack effective therapeutics. Finally, Retnla deficiency significantly accelerated the expulsion of *N. brasiliensis* from the gut. When viewed together, the combined results from all three models establish a critical immunoregulatory role for Retnla in helminth induced Th2-type immunity.

## Materials and Methods

### Ethics statement

All animal work was conducted according to relevant national and international guidelines.

### Mice, parasite infections

The Retnla^−/−^ mice [39] were generated with Velocigene technology [40] and backcrossed to the C57BL/6 background for 10 generations at the NIH. IL-13^−/−^, IL-4^−/−^, IL-4^−/−^/IL-13^−/−^, IL-13^−/−^/IFN-γ^−/−^, IFN-γ^−/−^, IL-4^−/−^/IL-13^−/−^/IFN-γ^−/−^, and IL-5^−/−^ mice were obtained from the NIAID Taconic contract. All mice were housed under specific pathogen-free conditions at the National Institutes of Health in an American Association for the Accreditation of Laboratory Animal Care approved facility. The NIAID animal care and use committee approved all experimental procedures. For infections, mice were percutaneously exposed via the tail with 30–35 cercariae of a Puerto-Rican Strain of *S. mansoni* (NMRI) that were obtained from infected *Biomphalaria glabrata* snails (Biomedical Research Institute, Rockville, MD). All mice were perfused at the time of sacrifice and worm and tissue egg burdens were determined. *Nippstrongylus brasiliensis* larvae (L3) were prepared as previously described [13]. WT and Retnla^−/−^ mice were inoculated through s.c. injection of 500 L3. On day 4, 7, and 12 after inoculation adult worms were enumerated from jejunum, eggs were counted in the feces, and lungs were collected for cytokine mRNA analysis. rRelma was obtained from Leinco Technologies, Inc. for the in vitro studies (product # R1140, St. Louis, MO).

### Histopathology and fibrosis

Pulmonary and hepatic granuloma measurements from schistosome-exposed mice were determined from histological sections stained with Wright's Giemsa or Picrosirius Red (Histopath of America, Clinton, MD). Peribronchiolar inflammation was also quantified in the lungs of *Nippstrongylus brasiliensis*-infected mice (arbitrary scale 1–4). Thirty granulomas per mouse were included in all analyses of schistosome granulomas. The number of schistosome eggs in the liver and the gut and the collagen content of the liver, as measured by hydroxyproline levels, were determined as previously described [13]. Specifically hepatic collagen was measured after hydrolysis of a 200-mg portion of liver in 5 ml of 6N HCl at 110°C for 18 h. The increase in hepatic hydroxyproline was positively related to egg numbers in all experiments and hepatic collagen is reported as the increase above normal liver collagen in micromoles per 10,000 eggs; (infected liver collagen – normal liver collagen)/liver eggs ×10^−4^ or micromoles per worm pair. At chronic time points, fibrosis is reported as total liver collagen per liver. The same individual scored all histological features and had no knowledge of the experimental design.

### β-galactosidase staining

All tissues were processed for β-galactosidase staining according to a protocol published previously [35]. Briefly, formalin fixed tissue samples were embedded in OCT compound (Tissue-Tek) and 8 µm sections were prepared on polylysine coated glass slides. Dried acetone fixed slides were stained in a LacZ staining solution overnight at 37°C and briefly counter stained with Diff Quick® thiazine dye solution (Boehringer).

### Purification and analysis of eosinophils

Eosinophils were isolated from the livers of 8–9 wk *S. mansoni*-infected C57BL/6 mice. Single-cell suspensions were prepared from liver homogenates following a collagenase I (40 µg/ml) and DNase I (2 µg/ml) digestion at 37°C for 45 min. The digested mixture was passed through a 100-µm cell sieve and washed in phosphate-buffered saline, and the leukocyte fraction was enriched over 38% isotonic Percoll. After the Percoll step, ammonium chloride and potassium bicarbonate lysing buffer was used to remove any contaminating red blood cells. Eosinophils were subsequently isolated with a MiniMACS column using the Siglec-F–phycoerythrin monoclonal antibody (MAb) (BD Biosciences-Pharmingen, San Diego, CA). The purity of the eosinophil fraction (95%) was determined by flow cytometry and confirmed microscopically by fast green staining (Sigma) of cytospin preparations using standard protocols [36]. Nonfractionated cells and Siglec-Fnegative and Siglec-F-positive fractions were used to prepare total RNA for realtime PCR analysis according to the RNeasy animal cell I protocol provided by the manufacturer.

### ELISA

Immulon 2HB plates (Thermo) were coated overnight with *S. mansoni* egg antigen (SEA) at 10 µg/ml in PBS. After blocking the plates with 5% nonfat dry milk (Carnation), sera were added at various dilutions from 1∶10 onwards. The following secondary antibody (Southern Biotech) was used to detect IgE —anti-IgE (23G3). sIL-13Rα2 was determined by ELISA. Briefly, total IL-13Rα2 were quantitated by coating Immulon 2HB plates with mouse IL-13Rα2 Affinity Purified Polyclonal Ab (R&D Systems, Minneapolis, MN) to capture the total amount of soluble IL-13Rα2 in the serum. Samples were compared against a serial-fold dilution of rmIL-13Rα2 Fc/chimera standard (R & D Systems, Minneapolis, MN). The sensitivity of the assay was approximately 98 pg/ml.

### RNA isolation and purification and real time polymerase chain reaction

Individual sample RNA (0.1 µg) was reverse-transcribed using Superscript II (Invitrogen, Carlsbad, CA) and a mixture of oligo (dT) and random primers. Real- time polymerase chain reaction (RT-PCR) was performed on an ABI Prism 7900 sequence detection system (Applied Biosystems, Foster City, CA). Relative quantities of mRNA for several genes was determined using SYBR Green PCR Master Mix (Applied Biosystems, Foster City, CA) and by the comparative threshold cycle method as described by Applied Biosystems for the ABI Prism 7700/7900 sequence detection systems. In this method, mRNAs for each sample were normalized to hypoxanthine guanine phosphoribosyl transferase (HPRT) mRNA amounts and then expressed as a relative increase or decrease compared with uninfected controls. Sequences for *hprt*, *Il4*, *Il5*, *Il13*, *Il25*, *Ifng*, *Ccl11*, *Muc5ac*, *and Gob5*were published previously [35].

### Statistics

Hepatic fibrosis (adjusted for egg number) decreases with increasing intensity of infection (worm pairs). Therefore, these variables were compared by analysis of covariance, using the logarithm of total liver eggs as the covariate and the logarithm of hydroxyproline content per egg. All other data was analyzed with Prism (Version 5; GraphPad). Data were considered statistically significant for *P* values less than 0.05, obtained with a two-tailed *t*-test.

## Supporting Information

Figure S1 Immunological characterization of Retnla^−/−^ mice. Flow cytometry of single-cell suspensions of homogenized thymus and spleen from naive Retnla^+/+^ and Retnla^−/−^ littermates. Lymphocytes are gated based on forward- and side-scatter parameters. Numbers in quadrants indicate percent among lymphocytes. Data are representative of two experiments with three mice per group.
(0.12 MB DOC)
