# Supplementary material for: Retnla (Relmα/Fizz1) Suppresses Helminth-Induced Th2-Type Immunity
Source: PLoS Pathog. 2009 Apr 17;5(4):e1000393. doi: 10.1371/journal.ppat.1000393 (PMC2663845; doi:10.1371/journal.ppat.1000393)
Supplement: Figure S1 — Immunological characterization of Retnla−/− mice. Flow cytometry of single-cell suspensions of homogenized thymus and spleen from naive Retnla+/+ and Retnla−/− littermates. Lymphocytes are gated based on forward- and side-scatter parameters. Numbers in quadrants indicate percent among lymphocytes. Data are representative of two experiments with three mice per group. (0.12 MB DOC) [file ppat.1000393.s001.doc]

**Supplemental Figure 1. Immunological characterization of Retnla^-/-^ mice.**

Flow cytometry of single-cell suspensions of homogenized thymus and spleen from naive Retnla**^+/+^** and Retnla**^-/-^** littermates. Lymphocytes are gated based on forward- and side-scatter parameters. Numbers in quadrants indicate percent among lymphocytes. Data are representative of two experiments with three mice per group.


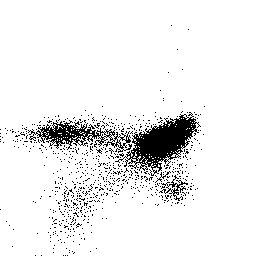


**4.56**

**84.4**

**8.67**

**2.4**

WT

Retnla^-/-^


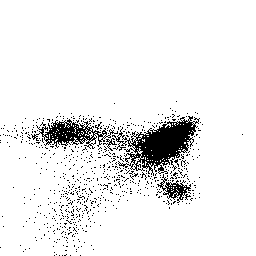


**6.43**

**82.5**

**8.81**

**2.26**


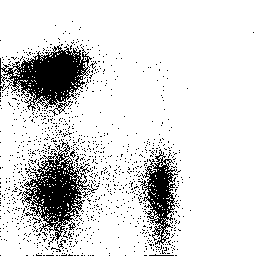


**15.1**

**0.12**

**56.1**

**28.6**


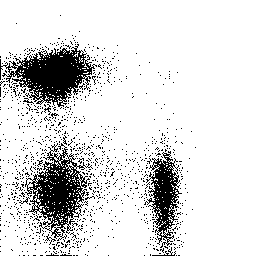


**16.2**

**0.15**

**55.5**

**28.2**


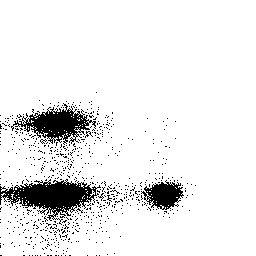


**16.4**

**0.095**

**23.1**

**60.4**


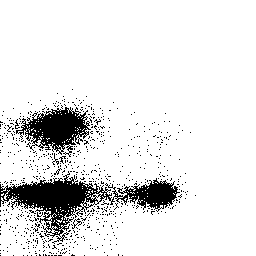


**15.1**

**0.21**

**23**

**61.7**


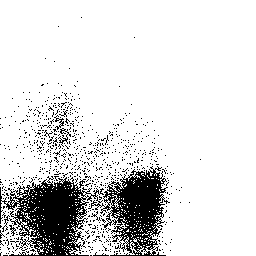


**38.3**

**0.74**

**3.28**

**57.7**


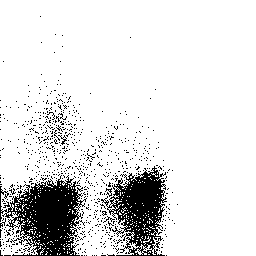


**36.4**

**0.53**

**2.74**

**60.3**


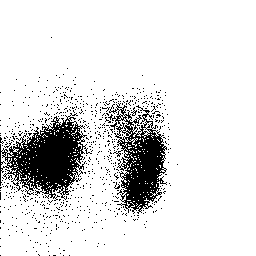


**13.8**

**23.6**

**52.8**

**9.7**


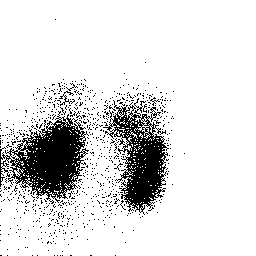


**14.6**

**23.4**

**51.2**

**10.8**

CD19

CD4

CD44

DX5

CD4

CD8

CD8

CD3

WT

Retnla^-/-^

**Thymus**

**Spleen**
